# High rates of virological failure and drug resistance in perinatally HIV-1-infected children and adolescents receiving lifelong antiretroviral therapy in routine clinics in Togo

**DOI:** 10.7448/IAS.19.1.20683

**Published:** 2016-04-27

**Authors:** Mounerou Salou, Anoumou Y Dagnra, Christelle Butel, Nicole Vidal, Laetitia Serrano, Elom Takassi, Abla A Konou, Spero Houndenou, Nina Dapam, Assetina Singo-Tokofaï, Palokinam Pitche, Yao Atakouma, Mireille Prince-David, Eric Delaporte, Martine Peeters

**Affiliations:** 1 Laboratoire de Biologie Moléculaire et d'Immunologie, Faculté des Sciences de la Santé, Université de Lomé, Lomé, Togo; 2 UMI 233, Institut de Recherche pour le Développement, INSERM U1175, Université de Montpellier, Montpellier, France; 3 Service de Pédiatrie CHU Sylvanus Olympio, Lomé, Togo; 4 Association Espoir pour Demain, Kara, Togo; 5 Espoir Vie Togo, Lomé, Togo; 6 Programme National de Lutte contre le Sida et les IST/Togo, Lomé, Togo; 7 Conseil National de Lutte contre le SIDA and Faculté des Sciences de la Santé, Lomé, Togo; 8 Département de Pédiatrie, Faculté des Sciences de la Santé, Université de Lomé, Lomé, Togo

**Keywords:** HIV, virological failure, drug resistance, children, antiretroviral treatment, Togo, Africa

## Abstract

**Introduction:**

Antiretroviral treatment (ART) has been scaled up over the last decade but compared to adults, children living with HIV are less likely to receive ART. Moreover, children and adolescents are more vulnerable than adults to virological failure (VF) and emergence of drug resistance. In this study we determined virological outcome in perinatally HIV-1-infected children and adolescents receiving ART in Togo.

**Methods:**

HIV viral load (VL) testing was consecutively proposed to all children and adolescents who were on ART for at least 12 months when attending HIV healthcare services for their routine follow-up visit (June to September 2014). Plasma HIV-1 VL was measured using the m2000 RealTime HIV-1 assay (Abbott Molecular, Des Plaines, IL, USA). Genotypic drug resistance was done for all samples with VL>1000 copies/ml.

**Results and discussion:**

Among 283 perinatally HIV-1-infected children and adolescents included, 167 (59%) were adolescents and 116 (41%) were children. The median duration on ART was 48 months (interquartile range: 28 to 68 months). For 228 (80.6%), the current ART combination consisted of two nucleoside reverse transcriptase inhibitors (NRTIs) (zidovudine and lamivudine) and one non-nucleoside reverse transcriptase inhibitor (NNRTI) (nevirapine or efavirenz). Only 28 (9.9%) were on a protease inhibitor (PI)-based regimen. VL was below the detection limit (i.e. 40 copies/ml) for 102 (36%), between 40 and 1000 copies/ml for 35 (12.4%) and above 1000 copies/ml for 146 (51.6%). Genotypic drug-resistance testing was successful for 125/146 (85.6%); 110/125 (88.0%) were resistant to both NRTIs and NNRTIs, 1/125 (0.8%) to NRTIs only, 4/125 (3.2%) to NNRTIs only and three harboured viruses resistant to reverse transcriptase and PIs. Overall, 86% (108/125) of children and adolescents experiencing VF and successfully genotyped, corresponding thus to at least 38% of the study population, had either no effective ART or had only a single effective drug in their current ART regimen.

**Conclusions:**

Our study provided important information on virological outcome on lifelong ART in perinatally HIV-1-infected children and adolescents who were still on ART and continued to attend antiretroviral (ARV) clinics for follow-up visits. Actual conditions for scaling up and monitoring lifelong ART in children in resource-limited countries can have dramatic long-term outcomes and illustrate that paediatric ART receives inadequate attention.

## Introduction

Of the more than three million children infected with HIV, 90% live in sub-Saharan Africa [1]. Over the last decade major advances have been made in almost every area of the fight against HIV, but progress for children and adolescents is falling behind, especially in resource-limited countries [2]. The increasing access to services for prevention of mother-to-child transmission (PMTCT) of HIV has reduced the number of new HIV infections among children. However, new HIV infections still occur: it is estimated that 190,000 children became infected in 2014 [1]. Without antiretroviral treatment (ART), about half of the children living with HIV die before the age of two years [3,4]. ART has been scaled up over the last decade but, compared to adults, children living with HIV are less likely to receive ART [2]. Moreover, in 2013, it was estimated that the majority of adolescents living with HIV in Africa were never diagnosed, were lost to follow-up or dropped out of treatment and care programmes [2]. AIDS-related deaths are also increasing among adolescents [2].

In resource-limited settings, in addition to the risk of acquired HIV resistance during PMTCT, the limited number of available paediatric-formulated antiretroviral drugs for the different age classes, the frequently observed poor adherence, the social environment, psychosocial factors and the absence of biological monitoring compromise ART efficacy in HIV-infected children and adolescents. These factors all make children and adolescents more vulnerable than adults to virological failure (VF) and the emergence of drug resistance [5–10]. Several studies have reported on the outcome of ART in children in Africa but only a few reports are available on long-term outcomes [10–15].

In Togo, scaling up and free access to ART started in 2007 in the capital city; they were expanded to semi-rural areas in 2008. The country adopted the WHO public health approach, and in 2013 at least 31,000 HIV-positive adults were receiving ART, which corresponds to almost 50% of those in need for ART according to the WHO guidelines from 2010 (CD4 count<350 cells/mm^3^). ART has also been scaled up among children, but only 2800 (23.3%) of the 12,000 children (<14 years) infected with HIV in Togo were receiving ART in 2013 [16]. We previously observed high levels of VF and drug resistance in adults on long-term first-line ART and among newly diagnosed infants (<18 months) [17,18]. The primary objective of this study was thus to determine virological outcome in perinatally HIV-1-infected children and adolescents receiving ART according to the national guidelines and attending routine HIV care centres in Togo.

## Methods

### Data and sample collection

Between June and September 2014, a free HIV viral load (VL) test was consecutively proposed for the purpose of the study to all perinatally HIV-1-infected children (aged<10 years) and adolescents (aged between 10 and 19 years) who were on ART for at least 12 months when attending private or public HIV healthcare services for their routine follow-up visit. HIV care centres were in the urban region of Lomé, the capital city, and in three semi-rural areas: the Maritime, Plateaux and Kara regions.

Demographic information was collected using a standardized questionnaire by the interviewer during sampling, and information on ART history was collected retrospectively on site from medical records. Whole blood was drawn by venepuncture on EDTA tubes (Becton Dickinson, Franklin Lakes, NJ, USA) and sent within four hours to the national reference laboratory (Laboratoire de Biologie Moléculaire et d'Immunologie, Faculté des Sciences de la Santé, Université de Lomé (BIOLIM/FSS-UL)) in Lomé and its satellite in Kara, especially for samples collected in the Kara sanitary region. Upon reception, five spots of 50 µL of whole blood were prepared for each patient onto (GE-Healthcare, PA, USA) Whatman 903 filter paper and dried at room temperature for three hours. Dried blood spots (DBS) were then placed individually in plastic bags and stored in a hermetic box containing silica desiccant at −20° until genotypic resistance testing in a WHO-accredited laboratory (Institut de Recherche pour le Développement, Montpellier, France). The remaining whole blood samples were centrifuged, and plasma was aliquoted and stored at −80°C for further HIV-1 RNA quantification. For the Maritime and Plateaux regions, DBS and plasma aliquots were prepared on site, stored at −20°C for a maximum of one week and subsequently transported by road in a cool box to the BIOLIM/FSS-UL laboratory in Lomé, where they were stored at −80°C for plasma and −20°C for DBS.

### Ethical considerations

The study was approved by the National Ethics Committee (CBRS/Togo) and Ministry of Health (N°752/2014/MS/CAB/DGS/DPLET/CBRS). Patients were recruited on a voluntary basis and, for all children, parents or legal guardians provided consent to participate in the study and were informed of the study objectives and procedures. A unique identifier was assigned to each sample and used throughout the study, ascertaining anonymity. Only physicians could establish the correspondence between this identifier and the patient.

### HIV-1 VL and genotypic drug resistance testing

Plasma HIV-1 VL was measured using the m2000rt Real Time HIV-1 assay (Abbott Molecular, Des Plaines, IL, USA) in the national reference laboratory (BIOLIM/FSS-UL) in Lomé. The lower limit of detection for the assay is 40 copies/ml (1.6 log copies/ml). Genotypic drug resistance testing was performed on all samples with plasma HIV-1 RNA load above 1000 copies/ml (3.0 log_10_ copies/ml). Nucleic acids were extracted with the NucliSENS easyMAG method (BioMerieux, Lyon, France) from two entire spots of 50 µl whole blood as previously described [19]. Protease and a 798-bp fragment from the reverse transcriptase (RT) region were amplified with the *in-house* protocol from the Agence Nationale de Recherche sur le Sida et les Hépatites en France (ANRS) (www.hivfrenchresistance.org/ANRS-procedures.pdf). PCR products were purified and directly sequenced using the BigDye Terminator v3.1 Cycle Sequencing Kit (Applied Biosystems, Carlsbad, CA, USA). The quality of all sequences was tested with the calibrated population resistance tool (www.cpr.stanford.edu/cpr.cgi). We used the ANRS interpretation algorithm, version 24 (www.hivfrenchresistance.org/2014/Algo-2014.pdf), to identify relevant drug resistance mutations (DRMs) and to predict drug resistance or possible resistance to antiretroviral drugs. We constructed phylogenetic trees with maximum likelihood methods implemented in PhyML to identify HIV-1 subtypes and circulating recombinant forms and to evaluate eventual epidemiologic links between samples [20]. The newly reported protease and reverse transcriptase sequences are available in GenBank [accession numbers: KT592383 to KT592507].

### Statistical analysis

Continuous variables were compared using the Wilcoxon rank-sum test, and comparisons between two categorical variables were made using the chi-square test and Fisher's exact test when appropriate.

## Results and discussion

### Characteristics of the study population at enrolment

A total of 283 perinatally HIV-1-infected children and adolescents aged 2 to 19 years who had been receiving ART for at least 12 months were consecutively included between June and September 2014. Patient characteristics are shown in Table 1. Overall, 189 were recruited at HIV health care centres in the urban area of the capital city Lomé, and 94 were from semi-rural areas – the Maritime (*n*=9), Plateaux (*n*=15) and Kara (*n*=70) regions. The male/female ratio was 141/142; 167 (59.1%) were adolescents and 116 (40.9%) were children. Twenty (7.1%) were less than five years old and among them only two were less than three years old. The median duration on ART for the entire study population was 48 months (interquartile range (IQR): 28 to 68 months). Median duration on ART increased with age categories: 21 months (IQR: 15 to 27) for children less than five years old and reaching 64 months (IQR: 52 to 86) for adolescents aged between 15 and 19 years. Mean duration on ART was only slightly lower in the semi-rural areas: 46 months (IQR: 26 to 68) versus 50 (IQR: 30 to 73) in Lomé.

**Table 1:** Characteristics of 283 perinatally HIV-1-infected children and adolescents studied

	Total	Female	Male
Characteristics	*n=*283	*n=*142	*n=*141
Median age, years (IQR)	10 (8 to 13)	10 (8 to 13)	10 (8 to 13)
Age categories, *n* (%)			
<5 years^a^	20 (7.1%)	7 (4.9%)	13 (9.2%)
5 to 10 years	127 (44.9%)	69 (48.6%)	58 (41.1%)
11 to 14 years	98 (34.6%)	50 (35.2%)	48 (34.1%)
15 to 19 years	38 (13.4%)	16 (11.3%)	22 (15.6%)
Orphaned, *n* (%)			
No	96 (33.9%)	49 (34.5%)	47 (33.3%)
Yes	89 (31.4%)	49 (34.5%)	40 (28.4%)
Unknown	98 (34.6%)	44 (31.0%)	54 (38.3%)
Geographical area, *n* (%)			
Urban (Lomé, capital city)	189 (66.8%)	90 (63.4%)	99 (70.2%)
Semi-rural	94 (33.2%)	52 (36.6%)	42 (29.8%)
Months on ART, median (IQR)	48 (28 to 68)	44 (27 to 65)	52 (30 to 70)
Months on ART per age category, median (IQR)			
<5 years	21 (15 to 27)	20 (15 to 29)	21 (14 to 27)
5 to 10 years	41 (27 to 57)	44 (28 to 60)	48 (27 to 63)
11 to 14 years	57 (33 to 76)	48 (31 to 76)	53 (36 to 76)
15 to 19 years	64 (52 to 86)	62 (39 to 84)	72 (58 to 98)
Months on ART per geographical area, median (IQR)			
Urban, Lomé, capital city	50 (30 to 73)	41 (27 to 62)	55 (36 to 76)
Semi-rural	46 (26 to 68)	45 (27 to 68)	46 (26 to 60)
ART regimen at inclusion, *n* (%)			
AZT+3TC+NVP/EFV	228 (80.6%)	119 (83.8%)	109 (77.3%)
ABC+3TC+NVP/EFV	16 (5.7%)	8 (5.7%)	8 (5.7%)
TDF+3TC+EFV	8 (2.8%)	4 (2.8%)	4 (2.8%)
AZT+3TC+ABC/TDF	3 (1.0%)	1 (0.7%)	2 (1.4%)
ABC+3TC+LPV/r or ATV/r	16 (5.7%)	4 (2.8%)	12 (8.5%)
AZT+3TC+LPV/r	7 (2.5%)	4 (2.8%)	3 (2.1%)
TDF+3TC+LPV/r or ATV/r	5 (1.7%)	2 (1.4%)	3 (2.1%)
Previous changes in ART regimen, *n* (%)			
No	138 (48.8%)	74 (52.1%)	64 (45.4%)
Yes	135 (47.7%)	63 (44.4%)	72 (51.1%)
Not available	10 (3.5%)	5 (3.5%)	5 (3.5%)
CD4 counts			
Available at sampling time, *n* (%)	163 (57.6%)	86 (60.6%)	77 (54.6%)
Median cell counts, cells/mm^3^ (IQR)	610 (347 to 947)	670 (302 to 905)	610 (412 to 1023)
PMTCT exposure, *n* (%)			
Yes	10 (3.5%)	3 (2.0%)	7 (5.0%)
No	165 (58.3%)	81 (57.0%)	84 (59.6%)
Not available	108 (38.2%)	58 (41.0%)	50 (35.4%)
Breastfeeding at a young age, *n* (%)			
Yes	159 (56.2%)	77 (54.2%)	82 (58.5%)
No	1 (0.3%)	1 (0.7%)	0 (0.0%)
Not available	123 (43.5%)	65 (45.1%)	58 (41.5%)

IQR, interquartile ratio; ART, antiretroviral therapy; AZT, zidovudine; 3TC, lamivudine; NVP, nevirapine; EFV, efavirenz; ABC, abacavir; TDF, tenofovir; LPV/r, boosted lopinavir; ATV/r, boosted atazanavir; PMTCT, prevention of mother-to-child transmission.

a Only two children were less than three years old.

For 228 (80.6%) patients, the current ART combination consisted of two nucleoside reverse transcriptase inhibitors (NRTIs), zidovudine (AZT) plus lamivudine, and one non-nucleoside reverse transcriptase inhibitor (NNRTI), nevirapine (NVP) or efavirenz (EFV). Only 28 (9.9%) were on a protease inhibitor (PI)-based regimen when included in the study, and the remaining 27 patients were on other Reverse transcriptase inhibitor (RTI) combinations (Table 1). The proportion of patients on PI regimens varied according to age categories; 4/20 (20%) for those under five years, 6/96 (6.3%) for those between five and ten years, 11/129 (8.5%) for those between 11 and 14 years and 7/38 (18.4%) between 15 and 19 years. Only 11 children were on first-line ART with PI, including the four (25%) children aged below five years. Although the WHO recommends first-line ART with a PI-based regimen for children aged less than three years, this was only the case for one of the two children in this age category. Only 7/28 (25%) of patients on PI regimens were from semi-rural areas. CD4 counts were available at sampling for 163 (57.6%), and median CD4 counts were 610 cells/mm^3^ (IQR: 347 to 977). VL was not done in routine follow-up and data on adherence were only occasionally noted in patient records. Only 10 (3.5%) patients had been exposed to PMTCT, for 165 (58.3%) no exposure was reported and information was lacking in the patient records for the remaining. For 159 (56.2%), breastfeeding at a young age was reported, but information on the ART status of the mothers was absent from patient records.

Retrospective data collection on ART history showed that a change in ART regimen occurred for 135 (47.7%) patients, mainly related to a change in the national guidelines (replacement of stavudine in 2010 by AZT) or replacement of NVP by EFV related to age (Table 1). Switches to a PI-containing regimen occurred in only 16 (5.7%). The proportion of treatment switches increased with age categories: 4/20 (20%) for participants under five years old, 39/96 (40.6%) for participants aged five to ten years, 66/129 (51.2%) for participants 11 to 14 years old and 26/38 (68.4%) for adolescents between 15 and 19 years old.

### High rates of VF among children and adolescents on lifelong ART

For only 102 (36%) patients, VL was below the detection limit of the VL assay used (i.e. 40 copies/ml), for 35 (12.4%) VL was between 40 and 1000 copies/ml and for 146 (51.6%; 95% CI 45.8 to 57.4) VL was above 1000 copies/ml (Table 2).

**Table 2:** Virological failure and drug resistance in 283 perinatally HIV-1-infected children and adolescents studied in Togo

Variables	*n*/*N* tested	(%)
VL (copies/ml)		
** **Undetectable (<40 copies/ml)	102/283	(36%)
** **40 to 1000	35/283	(12.4%)
** **>1000	146/283	(51.6%)
Median VL (log_10_ copies/ml), median (IQR)	4.55	(3.55 to 5.18)
*pol* gene sequenced	125/146	(85.6%)
Frequency of drug-resistant HIV^a^		
** **Resistance to NRTI only	1/125	(0.8%)
** **Resistance to NNRTI only	4/125	(3.2%)
** **Resistance to NRTI + NNRTI	110/125	(88.0%)
** **Resistance to NRTI + / − NNRTI + PI	3/110^b^	(2.7%)
Frequency of drug resistance to the different RT inhibitors^a^		
** **AZT	58/125	(46.4%)
** **D4T	58/125	(46.4%)
** **3TC/FTC	111/125	(88.9%)
** **ABC	19/125	(15.2%)
** **DDI	8/125	(6.4%)
** **TDF	3/125	(2.4%)
** **EFV	110/125	(88.0%)
** **NVP	113/125	(91.7%)
** **ETV	30/125	(25.0%)
** **RPV	82/125	(65.6%)
Predicted resistance to drugs of current ART regimen^a^		
** **No resistance	7/125	(5.6%)
** **1/3 drugs	10/125	(8.0%)
** **2/3 drugs	56/125	(44.8%)
** **2/3 drugs and possible resistance for 1/3	3/125	(2.4%)
** **3/3 drugs	49/125	(39.2%)

ART, antiretroviral therapy; VL, viral load; IQR, interquartile ratio; NRTI, nucleoside reverse transcriptase inhibitor; NNRTI, non-nucleoside reverse transcriptase inhibitor; PI, protease inhibitor; RT, reverse transcriptase; FTC, emtricitabine; AZT, zidovudine; D4T, stavudine; 3TC, lamivudine; ABC, abacavir; DDI, didanosine; ETV, Etravirine; TDF, tenofovir; EFV, efavirenz; NVP, nevirapine; RPV, rilpivirine.

a Drug resistance was predicted with version 24 of the algorithm by the Agence Nationale de Recherche sur le Sida et les Hépatites en France, July 2014. Only values for resistance were shown except when indicated;

b Protease sequences were only obtained for 110 patients.

According to the WHO criteria for resource-limited countries, patients are considered to have VF when VL is above 1000 copies/mL [21]. VF increased to some extent with the age of the children and time on ART, but not in significant proportions (*p*>0.05) (Table 3). Although the numbers were low, the VF rate was higher, 139/255 (54.5%), in patients on a current combination of RTIs versus 7/28 (25%) among those on PI-based regimens (*p*=0.003).

**Table 3:** Virological failure (VL > 1000 copies/ml) according to age category, months on ART and geographic origin in perinatally HIV-1-infected children and adolescents

	Virological failure rates (>1000 copies/ml), *N*/*N* (%)		
	
Characteristics	Total	Female	Male
Age categories (years)			
** **<5	9/20 (45.0%)	5/7 (57.1%)	4/13 (30.8%)
** **5 to 10	68/127 (53.6%)	42/69 (60.9%)	26/58 (44.8%)
** **11 to 14	47/98 (48.0%)	20/50 (40.0%)	27/48 (56.3%)
** **15 to 19	23/38 (60.5%)	11/16 (68.8%)	11/22 (50.0%)
Total	147/283 (51.9%)	78/142 (54.9%)	68/141 (48.2%)
Months on ART			
** **12 to 24	21/46 (45.6%)	10/24 (41.7%)	11/22 (50.0%)
** **25 to 36	34/61 (55.7%)	21/34 (61.8%)	13/27 (48.2%)
** **37 to 48	17/35 (48.6%)	12/20 (60.0%)	5/15 (33.3%)
** **49 to 60	24/46 (52.2%)	7/18 (38.9%)	17/28 (60.7%)
** **61 to 72	15/32 (46.9%)	9/17 (53.0%)	6/15 (40.0%)
** **>72	35/63 (55.6%)	19/29 (65.5%)	16/34 (47.1%)
Geographic origin		
** **Urban (Lomé, capital city)	93/189 (49.2%)	46/90 (51.1%)	47/99 (47.5%)
** **Semi-rural	53/94 (56.4%)	32/52 (61.5%)	21/42 (50.0%)

ART, antiretroviral therapy; VL, viral load.

Because the presence of family or close relatives can have an impact on adherence to therapy, we also compared VF rates between orphans and children with parents still alive, but no differences were observed: 51.1% (46/90) versus 54.6% (53/97), respectively (*p*>0.05). VF rates were slightly lower in Lomé than in the semi-rural clinics: 93/189 (49.2%) versus 53/94 (56.4%), respectively (*p*>0.05) (Table 3).

The overall VF rates observed among perinatally HIV-1-infected children in Togo are in general higher than those observed in previous reports in African countries. However, in these previous studies the median time on ART was often lower or fewer children were studied; for example 6% VF was observed after 3.3 years (IQR: 2.5 to 4.4) on ART in Kwazulu-Natal (South Africa) [22]; 16.7% with a median of 16 months on ART in Ghana [23]; 32, 53 and 55%, respectively, at 6, 12 and 24 months on ART in Senegal [15]; 15% in Cape Town (South Africa) after a median time of 2.4 years on a first-line ART protocol [24]; and 29% in Rwanda with a median duration of cART of 3.4 years [25]. In our study virological failure (VF) rates ranged from 45.6% (12 to 24 months on ART) to 55.7% (25 to 36 months on ART) and did not change significantly with time on ART or age (Table 3). This finding suggests the impact of multiple factors such as CD4 counts, VL or stage of disease at ART initiation. Alternatively, it could represent a bias because our study included only individuals still retained on ART and did not take into account the proportion of children who died or were lost to follow-up [26–28].

### High rates of drug resistance among children and adolescents on VF

Genotypic drug resistance testing was successful for 125/146 (85.6%) patients with VL >1000 copies/ml; 110 were sequenced on both reverse transcriptase and protease regions, and for 15 only reverse transcriptase sequences were obtained. Among the 125 patients with genotypes in RT, only seven were not predicted to be resistant in RT (Table 2) and only five (4%) had no DRMs yet. Among patients on VF, the overall proportion of drug-resistant strains was thus 94.4% (95% CI 90.4 to 98.4; 118/125); 110/125 (88%) were resistant to both NRTIs and NNRTIs; one (0.8%) was resistant only to NRTI; four (3.2%) to NNRTI only; and three harboured viruses resistant to RT and protease inhibitors. Among the patients harbouring PI drug-resistant viruses, exposure to PIs was only reported for one. Major PI mutations, L90M or M46L, were found in two patients without PI exposure and could represent a natural polymorphism [29], but M46I, I54V and V82A mutations were simultaneously present in one patient with 110 months on ART and with previous exposure to lopinavir boosted with ritonavir. Based on predictions of the ANRS drug resistance algorithm, 86% (108/125) of children and adolescents experiencing VF and successfully genotyped had either no effective ART or had only a single effective drug in their current ART regimen (Table 2). Nineteen, eight and three patients were also predicted to be resistant to abacavir, didanosine and tenofovir (TDF), respectively, although they had never been exposed to these drugs. Similarly, cross-resistance to the new NNRTIs, etravirine or rilpivirine, was observed in 30/125 (24%) and 82/125 (65.6%), respectively (Table 2).

**Figure 1:**
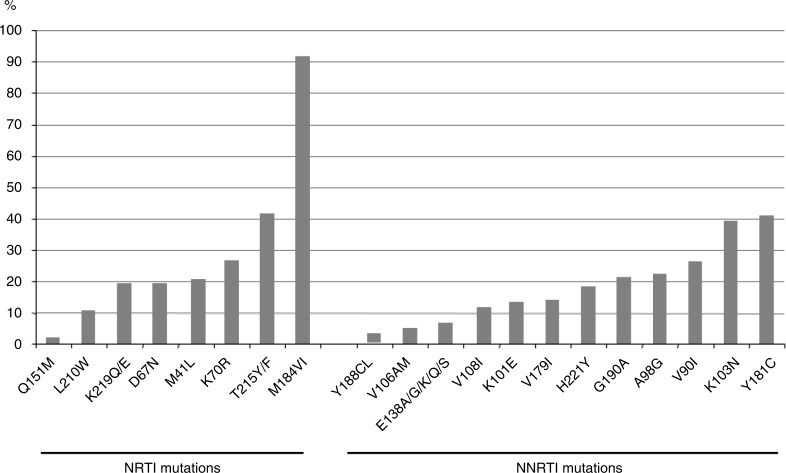
Proportion (%) of different nucleoside reverse transcriptase inhibitor and non-nucleoside reverse transcriptase inhibitor drug-resistant mutations.

Figure 1 shows the frequency of the different NRTI and NNRTI mutations. As expected, the most prevalent NRTI mutation was M184V/I (110/125; 91.7%), and thymidine analogue mutations (TAMs) were present in 68/125 (56.7%) patients. The number of TAMs increased with months on ART. Among subjects infected with TAM-containing viruses, 32/68 (47%) carried viruses with at least three TAMs. The multiple NRTI resistance Q151M complex that affects all NRTIs except TDF was found in two patients on ART for more than six years. The main NNRTI mutations were Y181C (*n*=49) and K103N (*n*=47).

The pattern of DRMs found in this study is consistent with the reports from several studies conducted in Africa [6,8,9]. The impact of DRMs acquired via PMTCT could not be evaluated in this study, but we previously reported that independent of perinatal antiretroviral (ARV) exposure, 60% of infants diagnosed with perinatal HIV infection in 2012 and 2013 were infected with drug-resistant strains [18]. Moreover, rates of drug resistance reached 80% in children receiving breast milk from mothers on ART [18]. These observations support the use of lopinavir-based first-line regimens in children as recommended by the WHO [21], especially with the recent national recommendations to implement lifelong ART for mothers. However, only 4 (20%) of the 20 children aged below five years were on a PI-based ART regimen in 2014 as recommended by the WHO, illustrating that these guidelines have not yet been implemented.

## Conclusions

Eliminating new paediatric HIV infections and improving the health and survival of mothers and their children in the context of HIV are part of the overall objectives of UNAIDS in 2015. Our study shows that 51.6% of perinatally infected children attending routine HIV care centres in Togo who had been on ART for more than 12 months (median 48 months) were experiencing VF and that 38% of the studied population were infected with multidrug-resistant strains. Despite certain limitations such as absence of information on how many children died of HIV or were lost to follow-up, our study provided important information on virological outcomes of lifelong ART in perinatally infected children and adolescents and illustrates that paediatric ART still receives inadequate attention. In addition, with scale-up of PMTCT programmes and implementation of lifelong ART for HIV-positive mothers, national programmes should make sure to use more robust first-line regimens, including PIs as recommended by the WHO [21]. More efforts are also needed to ensure early infant diagnosis and provide early access to ART for the paediatric population. The actual conditions for scaling up and monitoring lifelong ART in children in resource-limited countries have dramatic long-term outcomes. As for adults, access to routine VL monitoring is essential, in addition to the use of potent PI-based regimens and adapted formulations for the different age classes.
